# Attitudes and Behaviors toward traditional music among Chinese students: the role of individual, family, and school factors

**DOI:** 10.1371/journal.pone.0326009

**Published:** 2026-06-18

**Authors:** Jin Liu, Tian Li

**Affiliations:** School of Music and Dance, Guangzhou University, Guangzhou, Guangdong Province, China; PNG National Research Institute, PAPUA NEW GUINEA

## Abstract

**Background:**

Traditional music is vital for cultural identity, particularly among youth in China, yet factors influencing students’ engagement with it remain underexplored.

**Purpose:**

This study investigates the key factors affecting primary and secondary school students’ attitudes and behaviors toward traditional music in Guangdong Province, focusing on gender, grade level, parental support, and regional disparities.

**Methods:**

Utilizing a mixed-methods design, the research involved surveys and interviews with students, parents, and educators to gather comprehensive insights.

**Major Findings:**

The study revealed significant gender differences: female students displayed more positive attitudes and higher participation. Younger students exhibited greater enthusiasm, which declines with academic pressure. Parental involvement was identified as crucial, but the unexpected negative impact of parental artistic ability suggests more nuanced dynamics.

**Implications:**

These findings highlight the need for tailored music education policies that are inclusive and responsive to diverse student backgrounds, particularly in less developed regions.

**Conclusion:**

By understanding the multifaceted influences on students’ engagement, this study underscores the importance of fostering youth involvement in traditional music as a means of cultural preservation in an evolving educational landscape.

## 1. Introduction

Traditional Chinese music is a vital part of the nation’s cultural heritage, yet it faces significant challenges within the K-12 educational framework [1]. In Guangdong Province, disparities in music education are exacerbated by unequal resource allocation, a disconnect between policy and practice, and a declining student interest in traditional music. In an educational system that prioritizes standardized courses such as language arts and mathematics [2], the fundamental problem is the marginalization of traditional music teaching [3].

Despite existing policies, such as the National Medium- and Long-Term Education Reform and Development Plan (2010–2020) [4], which mandates improvements in cultural education, the implementation has been inconsistent. For instance, cities like Guangzhou have successfully integrated local musical traditions, like Cantonese opera, into school curricula [5]. In contrast, underdeveloped cities like Qingyuan and Yunfu grapple with significant gaps in teacher qualifications and educational resources, limiting students’ exposure to the rich cultural heritage of traditional music [6,7]. This disparity not only affects educational access but also diminishes students’ ability to engage with their heritage meaningfully. Existing literature has mainly focused on urban-rural divides, while ignoring the substantial intra-provincial disparities in the quality of music education between economically advanced and disadvantaged areas [8]. Recent studies highlight a lack of attention to critical factors such as parental involvement and curriculum quality [9], which are essential for fostering positive attitudes toward traditional music education [10]. Notably, younger students often develop an interest in music through sensory engagement, while older students require contextualization and real-life applications to sustain their motivation—needs that are often inadequately addressed in current curricula [11]. This study seeks to investigate the multifaceted factors systematically influencing primary and secondary school students’ attitudes toward traditional music in Guangdong Province. By examining regional disparities and their educational implications, this research aims to provide actionable recommendations for enhancing music literacy and supporting cultural transmission.

## 2. Literature review

### 2.1. Trends and issues in traditional music

Investigating the attitudes and behaviors of primary and secondary students toward traditional music carries significant implications for music education and cultural transmission. Recent research across various countries highlights a consistent trend: as students’ progress through grades, their engagement and motivation in music education decline [12,13], Finland [14], Malaysia [15], Uganda [16], Hungary [17], Russia [18], Turkey [19] and China [20,21]. Studies by Luo and Guan [22], Yanofsky [23], and Teng [24] illustrate that this decline can be framed through developmental psychology [25]. Piaget’s theory posits that students in the concrete operational stage (ages 7–11) show a stronger interest in music aligned with their sensory learning preferences [26]. In contrast, as they transition into the formal operational stage (ages 11 and above), the focus shifts toward abstract reasoning, often leading to reduced interest in music, exacerbated by rising academic pressures.

### 2.2. Key research themes

Research has identified three critical issues in traditional music education that influence students’ attitudes:

Cultural Identification: Students’ attitudes toward traditional music often reflect their cultural identity [27], with modernization and globalization contributing to declining interest in traditional forms [28].

Resource Accessibility: Disparities in educational policies and resource allocation hinder access to traditional music education [29], especially in under-resourced areas where ethnic music instruction is frequently overlooked [30,31].

Gender Dynamics: Female students typically exhibit greater participation rates in traditional music activities [32], aligning with societal perceptions of music as a feminine pursuit [33]. Conversely, male students may hesitate to participate due to fear of social marginalization [18,34].

These themes are interconnected, and attention to them is crucial for formulating effective educational strategies.

### 2.3. Research gaps in the Chinese context

While existing studies in China address various aspects of music education and cultural preservation [35], significant gaps persist. Current literature tends to focus on the relationship between familial economic status, school resource allocation, and music learning, often highlighting negative attitudes and low participation without exploring deeper underlying causes. However, it frequently falls short of exploring the deeper underlying causes and key influencing factors. Moreover, there is a lack of comparative analyses between more and less developed regions of China. This study seeks to fill these gaps by employing rigorous frameworks and statistical methods to analyze demographic differences, parental involvement, and school-related factors, with the ultimate goal of enhancing students’ attitudes toward traditional music education.

### 2.4. Factors influencing attitudes toward traditional music

Numerous factors synergistically shape students’ attitudes toward traditional music, encompassing individual differences, parental influences, and school-related variables. Understanding these factors is critical for educators and policymakers to devise effective strategies that promote positive attitudes toward traditional music education.

#### 2.4.1. Demographic differences.

Key demographic variables, particularly gender and grade level, significantly influence students’ attitudes toward traditional music:

Gender Perspectives: Studies indicate that female students typically show higher engagement levels in music activities [36,37]. This trend is rooted in societal expectations that associate music with femininity. Conversely, boys may suppress their interest in music due to fears of social marginalization.

Grade-Level Dynamics: Attitudinal differences are pronounced across grade levels. Younger students exhibit greater enthusiasm, while older students, often burdened by academic pressures, show diminished interest [22]. Acknowledging these dynamics can inform tailored pedagogical approaches and educational policies.

Examining the impact of gender and grade on students’ attitudes toward traditional music offers important insights for educators. These findings can help teachers customize their teaching methods and assist policymakers in developing more effective educational policies. Ultimately, this research supports the cultural sustainability of traditional music education.

#### 2.4.2. Personal factors.

Personal factors, such as self-concept and interest levels [38], play a crucial role in shaping attitudes toward traditional music[39].

Cognitive Regulation of Self-Concept: An individual's self-concept significantly influences receptivity to traditional music [40]. Research indicates that a positive self-concept enhances participation through pathways like metacognitive frameworks for musical understanding and sustained behavioral motivation [11,41].

Socialized Development of Interest: Bourdieu's habitus underscores that disparities in cultural capital impact students’ musical engagemen. Students from economically developed areas possess more stable engagement habits due to systematic investments in arts education. In contrast, those in resource-limited environments face challenges that inhibit interest [10,42].

#### 2.4.3. Parental factors.

Parental engagement in music education significantly impacts students’ attitudes through several mechanisms:

Dynamic Engagement: Parental involvement in music activities enhances children's cultural identification and intrinsic motivation [41,43].

Instrumental Rationality: As families approach music education from a utilitarian standpoint, this perspective can create cognitive dissonance between technical mastery and cultural appreciation [44].

#### 2.4.4. School factors.

School-related factors [45], particularly institutional constraints and resource allocation, are critical [46]:

Curriculum Implementation: Schools that effectively integrate traditional music into their curricula foster greater cultural agency among students [47]. However, exam-oriented systems often prioritize core subjects over arts education, leading to tokenistic inclusion of music [22].

Resource Distribution: The inequitable distribution of educational resources creates barriers for disadvantaged students, amplifying cultural discontinuities [48].

### 2.5. The regional ecology of traditional music education

This study will focus on three locations in Guangdong Province—Guangzhou, Yunfu, and Heyuan—serving as comparative case studies. Guangzhou, as an economic and cultural hub, demonstrates a robust music education system supported by significant investments in the arts. In contrast, Yunfu and Heyuan face substantial limitations, including teacher shortages and inadequate resources. Exploring these regional disparities allows for a deeper understanding of traditional music transmission and the socio-political dimensions of music education policy.

## 3. Study Purpose and Research Questions

### 3.1. Study purpose

The purpose of this study is to investigate the complex determinants influencing traditional music education in Guangdong Province, China. By focusing on three cities—Guangzhou, Yunfu, and Heyuan—representing varied economic conditions, the study aims to elucidate how regional economic disparities affect educational quality and access to traditional music education. It seeks to understand the interplay of structural factors, individual attributes, familial cultural capital, and institutional practices in shaping students’ attitudes toward traditional music.

### 3.2. Research questions

1. Do gender and grade level act as moderators affecting music-related factors at personal, parental, and school levels?
2. What are the developmental differences in the processes of music attitude formation across various educational stages?
3. What disparities exist in curriculum implementation efficacy and the quality of activity provision between developed and underdeveloped regions?
4. Is there a dose-response relationship between the intensity of parental support and the connections between traditional music attitudes and behaviors?
5. How do regional, grade, gender, school, and individual factors jointly influence the formation of cultural identity through multilevel cross-classified modeling (MLCM)?

## 4. Materials and methods

(1) Socioeconomic contexts of research sites

Data collection spanned from December 20–27, 2024.

This study focused on Guangzhou, Yunfu, and Heyuan in Guangdong Province as gradient comparison samples. As the core cultural and economic hub of Lingnan culture, Guangzhou boasts a robust music education system with a professional teacher coverage of 98.6% and per-student arts funding 2.3 times the provincial average [48]. In contrast, Yunfu and Heyuan face significant resource limitations, with a 41.5% shortage of professional teachers and per-student instrument availability at only 20% of Guangzhou's levels. These disparities provide valuable insights into regional differences in the transmission of traditional music.

(2) Policy-practice tensions in curriculum implementation

The Compulsory Education Curriculum Framework (2022 Edition) [50] mandates modular music curricula across K-12, integrating local musical heritage into school development [49]. Findings by Luo and Guan [22] reveal a gap in policy implementation, with urban schools fully adopting the curriculum while under-resourced regions struggle due to inadequate staffing and funding.

(3) Research design and theoretical framework

Stratified random sampling was employed in the study, constructing a tripartite analytical framework encompassing economic capital (GDP tiers), cultural capital (traditional music curriculum implementation), and habitus capital (family arts participation index). Through questionnaire surveys (N = 5,027), the study investigated:

1. The impact of economic gradients on traditional music access via resource redistribution;
2. The interplay between institutional curricula and self-directed learning in shaping attitudes;
3. Variations in cultural capital transmission efficiency across individual, family, and school contexts.

### 4.1. Participants and procedures

A three-stage stratified random sampling strategy categorized Guangzhou (developed), Yunfu (underdeveloped), and Heyuan (underdeveloped) based on GDP. A total of 5,027 valid cases were included in the final sample, ensuring a sufficient sample size for statistical analysis and representation of the varying regional contexts.

Inclusion Criteria: Participants were K-12 students across all grade levels (primary, middle, and high schools). Efforts were made to balance gender representation for sample demographic diversity.

This structured selection approach ensured a comprehensive and representative sample, facilitating robust analysis of factors influencing traditional music education across different socioeconomic contexts in Guangdong Province. Additionally, trained research assistants administered standardized questionnaires to systematically capture relevant factors.

Table 1 summarizes demographic characteristics of the 5,027 students, showing significant representation across geographical regions, gender, and grade levels.

**Table 1:** Descriptive Statistics for Demographic Characteristics.

Variables	Total (n = 5027)
Region, n (%)	
Guangzhou	1256 (24.99)
Heyuan	2407 (47.88)
Yunfu	1364 (27.13)
Gender, n (%)	
Male	2165 (43.07)
Female	2862 (56.93)
Grade, n (%)	
Primary school	1212 (24.11)
Junior high school	2626 (52.24)
High school	1189 (23.65)

### 4.2. Students’ attitudes and perceptions toward traditional music

A mixed-methods instrument was developed, incorporating the following dimensions:

- Attitudinal dimensions:5-point Likert scales for interest and cultural identity.
- Cognitive dimensions: Scenario-based assessments for knowledge mastery.

Behavioral intentions: Semantic differential scales for participation willingness.

#### 4.2.1. Personal factors.

Measurements were based on Petersen’s [11] self-efficacy theory and Hash’s [10] interest development model:

- Cognitive: 7-point Likert scales for musical self-concept (e.g., “I can accurately identify the unique rhythms of traditional music”);

Behavioral: Composite indices for course engagement (0–100 scale).

#### 4.2.2. Parental factors.

As assessed through Kinney’s [48] and Zdzinski’s [47] frameworks:

- Tangible support: Measured by practice frequency and space;

Symbolic value transmission: Parental expectations and encouragement via 7-point Likert scales [50].

#### 4.2.3. School factors.

Evaluated through student access to traditional music:

- **Curriculum Intensity**: Weekly course density and instruction continuity.
- **Activity Breadth**: Scope of elective offerings and annual incidence of school-sponsored traditional music activities (Table 2).

**Table 2:** Summary of Measurement Tools.

Construct	Measurement Tool	Description
Attitudinal Dimensions	5-Point Likert Scale	Measures interest and enjoyment towards traditional music; evaluates value judgments on cultural identity and modern adaptability.
Cognitive Dimensions	Scenario-Based Assessments	Evaluates knowledge mastery, including musical identification and historical context specific to traditional music.
Behavioral Intentions	Semantic Differential Scales	Assesses willingness to participate in music activities and the intent to share traditional music.
Musical Self-Concept	7-Point Likert Scale	Measures students’ confidence in their ability to identify rhythms and reproduce melodies of traditional music.
Parental Involvement	Composite Indices	Assesses levels of tangible support (frequency of practice involvement) and symbolic support (par-ental expectations and encouragement).
Activity Breadth	Structured School Music Opportunity Items	Assesses the scope of elective courses dedicated to traditional music and the annual incidence of school-sponsored traditional music events.
Curriculum Intensity	Weekly Course Density	Evaluates the number of music classes attended per week and the continuity of instruction in traditional music.

### 4.3. Future directions

Future efforts should focus on developing assessment tools for diagnosing resource gaps, creating multidimensional training models, and incorporating digital technologies to ameliorate institutional constraints.

### 4.4. Structural equation modeling framework

To systematically investigate the determinants of Chinese students’ attitudes and behaviors toward traditional music, a multilevel structural equation model (SEM) was developed. The model hierarchically prioritizes individual agency, familial socialization, institutional practices, and macroeconomic constraints, aligning with the “micro-to-macro” theoretical paradigm in educational research.

Note: In this study, “parents” refer to both parents or the primary caregivers as reported in the student questionnaires. Their involvement and behaviors are treated as proxy variables for the “family” level, used to analyze the influence of the family on children's traditional music education. This approach maintains consistency in terminology while clarifying the operational definition of the concept.

## 5. Results

### 5.1. Research question 1: Moderating effects of gender and grade

Independent samples t-tests and ANOVAs were performed to assess differences across genders(Table 3). Results indicated no significant differences in the quantity of traditional music curricula (t = 0.433, p = 0.665). However, female students showed significantly more positive attitudes toward music classes (t = −7.965, p < .001), higher self-concept (t = −2.735, p < .001), greater parental involvement (t = −5.462, p < .001), and more favorable parental attitudes (t = −8.178, p < .001).

**Table 3:** Differences in Traditional Music Curriculum and Student Attitudes by Gender.

Measure	t-value	p-value	Significance
Quantity of Traditional Music Curricula	0.433	0.665	Not Significant
Attitude Toward Music Classes	−7.965	< 0.001	Significant
Self-Concept	−2.735	< 0.001	Significant
Parental Involvement	−5.462	< 0.001	Significant
Parental Attitudes	−8.178	< 0.001	Significant

Independent samples t-tests and analysis of variance (ANOVA) were employed to assess significant differences in variables across groups. The t-tests compared mean differences between gender groups, while ANOVA examined differences across grade-level groups, with statistical significance set at p < .05.

As shown in Table 4, no significant gender differences were found in the quantity of school-based traditional music curricula and activities (t = 0.433, p = .665). Significant gender disparities emerged in students’ overall attitudes toward music classes, self-concept assessments, parental involvement, and parental attitudes. Female students demonstrated significantly more positive attitudes toward school music programs (t = −7.965, p < .001) and higher self-concept scores (t = −2.735, p < .001), indicating stronger self-identification among female participants. Additionally, female students exhibited significantly greater parental involvement (t = −5.462, p < .001) and more favorable parental attitudes (t = −8.178, p < .001) compared to male students.

**Table 4:** One-way ANOVA.

Variables	Total (n = 5027)	Male (n = 2165)	Female (n = 2862)	Statistic	*P*
elective traditional music courses and activities, mean ± sd	4.48 ± 2.41	4.50 ± 2.40	4.47 ± 2.41	t = 0.433	0.665
attitude, mean ± sd	5.36 ± 1.48	5.16 ± 1.55	5.50 ± 1.40	t = −7.965	<0.001
self-concept, mean ± sd	5.14 ± 1.44	5.07 ± 1.47	5.20 ± 1.42	t = −2.735	<0.001
parental involvement, mean ± sd	3.09 ± 2.34	2.88 ± 2.44	3.25 ± 2.26	t = −5.462	<0.001
parental attitude, mean ± sd	3.50 ± 2.44	3.18 ± 2.52	3.75 ± 2.35	t = −8.178	<0.001

### 5.2. Research question 2: Developmental differences in music attitude formation

Grades significantly influenced students’ perceptions regarding music education (Table 5). Lower-grade students exhibited higher engagement levels in traditional music courses and activities, as well as stronger self-concept and attitudes towards music (p < .001).

**Table 5:** MANOVA and Univariate F-test Results.

Variables	Total (n = 5027)	Primary School (n = 1212)	Junior High School (n = 2626)	High School (n = 1189)	Statistic
Elective Traditional Music Courses and Activities, mean ± sd	4.48 ± 2.41	4.62 ± 2.24	4.66 ± 2.43	3.95 ± 2.46	F = 38.856
Attitude, mean ± sd	5.36 ± 1.48	5.62 ± 1.43	5.35 ± 1.45	5.11 ± 1.54	F = 36.000
Self-Concept, mean ± sd	5.14 ± 1.44	5.46 ± 1.44	5.11 ± 1.43	4.87 ± 1.42	F = 39.581
Parental Involvement, mean ± sd	3.09 ± 2.34	3.55 ± 2.45	3.07 ± 2.30	2.68 ± 2.23	F = 42.105
Parental Attitude, mean ± sd	3.50 ± 2.44	4.01 ± 2.52	3.48 ± 2.41	3.04 ± 2.32	F = 49.069

Table 6 demonstrates significant grade-level effects: primary students showed higher musical attitudes (M = 5.62 vs. 5.11; F = 36.000, p < .001), stronger self-concept (F = 39.581, p < .001), and greater parental involvement (F = 42.105–49.069, p < .001) than high school cohorts. Traditional music clubs negatively correlated with musical attitudes (r = −0.220), self-concept (r = −0.205), and parental involvement (r = −0.226) (all p < .01), whereas music class frequency positively associated with self-concept (r = 0.104) and parental attitudes (r = 0.100). Class cancellations had negligible impact (r = 0.070). Traditional curriculum exposure strongly predicted positive attitudes (r = 0.539) and behaviors (r = 0.603), with self-concept and attitudes showing bidirectional reinforcement (r = 0.539).

**Table 6:** Descriptive statiatics and pearson correlations for the measures.

	α	range	mean	sd	1	2	3	4	5	6	7	8	9	10	11	12	13
traditional music groups availability	–	1-2	10.33	00.47	1												
weekly instructional frequency	–	1-4	20.12	00.52	−0.076**	1											
music class displacement	–	1-6	30.05	00.68	−00.008	0.022	1										
elective traditional music courses and activities	0.766	1-8	40.48	20.41	−0.138**	0.079**	0.041**	1									
self-concepts	–	1-7	50.14	10.44	−0.205**	0.104**	0.070**	0.030	1								
attitude	0.916	1-7	50.36	10.48	−0.220**	0.076**	0.021	0.020	0.539**	1							
behavior	0.792	1-7	100.29	20.73	−0.222**	0.085**	0.074**	−0.011	0.537**	0.451**	1						
cognition	0.937	1-7	40.14	10.50	−0.191**	0.052**	0.068**	−0.038**	0.425**	0.313**	0.603**	0.521**	1				
parental artistic ability	–	1-2	10.27	00.44	0.207**	−00.02	−00.02	−00.014	−0.165**	−0.154**	−0.253**	−0.246**	−0.275**	1			
parental educational background	0.849	1-7	70.62	20.33	−0.088**	0.077**	−0.084**	00.002	0.165**	0.160**	0.078**	0.095**	0.086**	−0.048**	1		
parental involvement	0.846	1-7	30.09	20.34	−0.226**	0.112**	0.037**	−0.031*	0.430**	0.291**	0.385**	0.274**	0.360**	−0.168**	0.225**	1	
parental attitude	0.783	1-7	30.50	20.44	−0.232**	0.100**	00.023	−0.032*	0.512**	0.344**	0.368**	0.295**	0.348**	−0.178**	0.245**	0.890**	1

Parental involvement mediated key outcomes: self-concept (r = 0.430), attitudes (r = 0.512), and behaviors (r = 0.385) (all p < .01). These findings necessitate developmental-stage pedagogy and family-institutional partnerships for sustainable music education.

### 5.3. Research question 3: regional disparities in curriculum quality

Developed regions had lower music class displacement and offered more elective courses as compared to underdeveloped regions (Table 7), demonstrating significant institutional disparities in the efficacy of traditional music education delivery.

**Table 7:** One-way ANOVA Results.

Variables	Total (n = 5027)	Developed City (n = 1256)	Underdeveloped City (n = 3771)	Statistic	P
Music Class Displacement, mean ± SD	4.32 ± 1.56	4.12 ± 1.63	4.39 ± 1.53	t = −5.040	<.001
Number of Elective Courses in Chinese Traditional Music, mean ± SD	4.66 ± 2.82	4.81 ± 2.71	4.61 ± 2.85	t = 2.194	<.028
Frequency of Traditional Music Events, mean ± SD	4.30 ± 2.94	4.25 ± 2.88	4.32 ± 2.97	t = −0.691	0.489

### 5.4. Research question 4: Parental Involvement and dose-response relationships

Parental involvement significantly influences students’ attitudes and behaviors towards traditional music (Table 8). While active parental engagement enhances musical appreciation, parental artistic competence shows a negative relationship, possibly due to compensatory strategies in families with less formal music training.

**Table 8:** Logistic Regression Model for Attitude and Action.

Variable	β	SE	t	p	95% CI
Region	0.144	0.032	4.498	<0.001	0.081, 0.207
Gender	0.071	0.049	1.449	0.147	−0.025, 0.167
Grade	0.188	0.035	5.304	<0.001	0.119, 0.257
School Music Groups	−0.241	0.056	−4.333	<0.001	−0.350, −0.132
Elective Traditional Music Courses and Activities	−0.009	0.010	−0.884	0.377	−0.029, 0.011
Attitude	0.347	0.021	16.847	<0.001	0.307, 0.387
Self-Concept	0.367	0.021	17.197	<0.001	0.325, 0.408
Parental Artistic Ability	−0.512	0.058	−8.865	<0.001	−0.625, −0.398
Parental Involvement	0.118	0.020	6.070	<0.001	0.080, 0.157
Parental Attitude	0.188	0.024	8.015	<0.001	0.142, 0.235

### 5.5. Research question 5:influences from multiple factors

Results reveal that regional, grade, gender, school, and personal factors collectively influence students’ cultural identity formation through engagement with traditional music. Developed regions show more effective music education policies, while parental involvement remains a powerful predictor of students’ music attitudes and behaviors.

## 6. Discussion

This study systematically investigates the factors influencing primary and secondary school students’ attitudes and behaviors toward traditional music in Guangdong Province, China. The discussion is organized around five core research questions, each of which is directly addressed to clarify findings and recommendations.

The data revealed significant gender differences in music attitudes, with female students demonstrating stronger positive attitudes and higher self-concept scores than male students. This aligns with previous studies indicating that gender socialization influences musical participation [42]. Boys often suppress their musical interests due to societal norms that contextualize music as less masculine. The findings suggest that educational settings should adopt gender-responsive curricula that address these stereotypes, encouraging greater participation from male students.

The study highlights a nonlinear trajectory in musical engagement, where younger students exhibit greater enthusiasm and self-concept in music [51]. As students progress academically, increased pressures and a shift toward abstract reasoning diminish their engagement [52]. This phenomenon underscores the need for developmentally sensitive curricula [53]. The presence of supportive environments in earlier grades should be leveraged to sustain engagement as students transition to higher grades.

Parental involvement emerged as a vital predictor of students’ attitudes and behaviors toward traditional music, reinforcing existing literature on the impact of family engagement in education [43,54]. However, the negative correlation between parental artistic ability and student engagement suggests that families lacking formal training may adopt compensatory strategies, emphasizing quantity of involvement over quality [55]. Future studies should explore how parents’ perceived competencies influence their support and students’ musical identity formation.

The interaction between regional factors, grade level, and gender significantly shaped students’ cultural identities through their engagement with traditional music. Developed areas exhibited stronger institutional frameworks that support music education, while disadvantaged regions lack the necessary infrastructure [42]. This discrepancy highlights the need for holistic educational policies that integrate community resources and familial input to foster cultural engagement, promoting sustainable music education across all demographics.

## 7. Recommendations for Future Research

Further studies should explore the causal mechanisms behind the observed contradictions in parental roles and delve deeper into the implications of regional disparities in music education quality. Some prioritized recommendations are stated below:

***Develop Gender-Responsive Curricula*** to address gender biases in music education to enhance male participation.

Enhance Resource Allocation: Policies that ensure equitable distribution of music education resources across regions should be implemented.

*Foster Family Engagement*: Workshops should be conducted to educate families about the cultural significance of traditional music, enhancing their involvement in music education.

## 8. Limitations

This study had several limitations that should be considered in future research. First, the regional concentration of the sample limited the generalizability of the findings. Although the study highlighted disparities in resource allocation across different regions, it did not fully address the structural challenges faced in rural and remote areas. Second, the cross-sectional design, while effective in capturing associations between variables, restricted causal inference—especially concerning the dynamic relationships between parental roles and student engagement. Third, the reliance on self-reported data may introduce bias, particularly on sensitive topics such as familial cultural capital and gender perceptions. Moreover, the study predominantly focused on the context of traditional music education in China, where the conclusions might be influenced by culturally specific factors. For example, the influence of Confucian family norms and collectivist educational orientations on gender roles and intergenerational transmission may vary when compared to other cultural systems. Future research that incorporates cross-cultural perspectives (e.g., comparing cultural capital transmission in traditional music education across China, South Korea, and the United States) could help clarify universal mechanisms and culture-specific pathways, ultimately enhancing the theoretical breadth and depth of the framework.

## 9. Conclusion and implications

This study highlights the critical role of gender, grade level, familial involvement, and regional disparities in shaping the attitudes and behaviors of primary and secondary school students toward traditional music in Guangdong Province, China. Given below are recommendations based on the findings: (i) **Implement Gender-Responsive Curricula**: To encourage greater participation from male students and promote a more inclusive music education environment. (ii) **Enhance Resource Sharing Mechanisms**: Policymakers should focus on equitable distribution of educational resources, particularly in underdeveloped regions, to ensure all students have access to quality music education. (iii) **Foster Parental Engagement**: Developing programs that educate families about the cultural significance of traditional music can strengthen parental involvement, thus enhancing students’ motivation and engagement. Despite the valuable insights offered by this research, limitations exist regarding its regional focus and potential biases from self-reported data. The participants in this study were limited to specific demographics, potentially overlooking the experiences of marginalized groups outside the primary focus. Expanding the sample to include diverse populations would enrich the understanding of music participation dynamics. While the study captures cross-sectional data, a longitudinal approach would provide insights into how music participation and related socialization processes evolve, especially across different educational stages. The influence of external sociopolitical factors, including policy changes and economic conditions, was not fully explored. Investigating these elements could reveal additional layers of complexity affecting music education participation. Although the study proposes AI/VR-mediated pedagogies, further research is needed to evaluate their effectiveness in diverse educational settings and with various learner groups. By addressing these limitations in future research, we can enhance our understanding of traditional music education and its interplay with sociocultural factors, leading to more inclusive and effective educational strategies. Future studies should utilize longitudinal designs and explore diverse educational contexts to enrich the understanding of traditional music education across China. The focus on specific developed regions may limit the generalizability of the findings. Future studies should integrate critical rural pedagogy frameworks to explore how regional differences impact music education more comprehensively. This multi-faceted approach aims to promote cultural sustainability and enrich students’ musical identities, ultimately contributing to the preservation of traditional music within a rapidly modernizing educational landscape.

Building upon the acknowledged limitations—including regional concentration, cross-sectional design, and cultural specificity—future research should adopt multi-sited longitudinal designs that incorporate critical rural pedagogy frameworks and cross-cultural comparisons (e.g., across East Asian and Western contexts) to validate and refine the proposed mechanisms.

## Supporting information

S1 Appendix Appendix. (PDF)
