# Correction: Attitudes and Behaviors toward traditional music among Chinese students: the role of individual, family, and school factors

**DOI:** 10.1371/journal.pone.0360159

**Published:** 2026-10-06

**Authors:** The PLOS One Staff

The authors are listed out of order. Please view the correct author order and citation here:

Tian Li, Jin Liu

Li T, Liu J (2026) Attitudes and Behaviors toward traditional music among Chinese students: the role of individual, family, and school factors. PLoS One 21(6): e0326009. https://doi.org/10.1371/journal.pone.0326009

The publisher apologizes for the error.
