# Supplementary material for: Attitudes and Behaviors toward traditional music among Chinese students: the role of individual, family, and school factors
Source: PLoS One. 2026 Jun 18;21(6):e0326009. doi: 10.1371/journal.pone.0326009 (PMC13278576; doi:10.1371/journal.pone.0326009)
Supplement: S1 Appendix — (PDF) [file pone.0326009.s001.pdf]

Appendix

Model specification:

Attitudes/Behaviors=β1(Personal self-concept) + β2(Family cultural capital) + β3(School curriculum intensity) + β4(Economic gradient) + ζ

Operational definitions:

Personal self-concept: A latent construct measured by:

Rhythm Identification: "I can accurately identify traditional rhythms" (7-point Likert, Q5)

Melodic reproduction: Scenario-based assessment of folk song replication accuracy (Q6)

Family cultural capital: A latent construct indexed by:

Tangible support: Weekly parental observation frequency during practice (0–7 scale, Q7)

Symbolic encouragement: "I expect my child to master traditional music" (7-point Likert, Q8)

School curriculum intensity: A composite observed variable:

Weekly course density (0–3 classes, ordinal)

Instructional continuity (5-point cancellation frequency scale)

Economic gradient: Ordinal variable (Guangzhou=3, Yunfu=2, Heyuan=1) derived from 2023 Guangdong statistical yearbook.

Attitudes/behaviors: A second-order latent variable:

Attitudes: Interest (Q1) and value judgment (Q2) (5-point Likert)

Behaviors: Participation frequency (Q3) and dissemination willingness (Q4)

Theoretical rationale:

Individual agency: Personal self-concept captures students' self-perceived competence in rhythm identification and melodic reproduction, which drives proactive engagement with traditional music.

Familial influence: The dual mechanisms of behavioral support (tangible practice accompaniment) and symbolic encouragement (value internalization) reflect the intergenerational transmission of musical cultural capital.

Institutional mediation: School curriculum intensity operationalizes structural educational inputs, where course density and continuity determine systematic exposure to traditional music.

Structural constraint: Regional economic gradients manifest through resource allocation disparities, creating institutional barriers to equitable musical education.

Analytical protocol:

Data preparation:

Missing data (<2.1%) handled via Full Information Maximum Likelihood (FIML)

Categorical variables scaled using robust weighted least squares (WLSMV)

Measurement validation:

Confirmatory factor analysis (CFA) for latent constructs:

Personal self-concept: χ^2/df = 1.24, RMSEA = 0.021, CFI = 0.988

Family cultural capital: CR = 0.89, AVE = 0.67

Second-order CFA for Attitudes/Behaviors: χ^2/df=2.17, SRMR = 0.033

Structural model testing:

Maximum likelihood estimation with robust standard errors (MLR)

Significance testing via bias-corrected bootstrap (1,000 resamples)

Model fit indices:

χ^2/df=1.93, RMSEA=0.031 (90% CI: 0.026–0.036), CFI=0.963, TLI=0.957

Standardized residuals: 92.7% within ±1.96 threshold
